# Supplementary material for: Mitigation of Galvanic Corrosion in Bolted Joint of AZ31B and Carbon Fiber-Reinforced Composite Using Polymer Insulation
Source: Materials (Basel). 2021 Mar 29;14(7):1670. doi: 10.3390/ma14071670 (PMC8036256; doi:10.3390/ma14071670)
Supplement: Supplementary file 1 [file materials-14-01670-s001.pdf]

materials

MDPI

Supplementary Materials

Mitigation of Galvanic Corrosion in Bolted Joint of AZ31B and Carbon Fiber-Reinforced Composite Using Polymer Insulation

Jiheon Jun [1,*], Yong Chae Lim [1,*], Yuan Li [1], Charles David Warren [2,†] and Zhili Feng [1]

[1] Materials Science and Technology Division, Oak Ridge National Laboratory, Oak Ridge, TN 37831 , USA; liy5@ornl.gov (Y.L.); fengz@ornl.gov (Z.F.)
[2] Energy and Transportation Science Division, Oak Ridge National Laboratory, Oak Ridge, TN 37831, USA; cdavewarren@tds.net
* Correspondence: junj@ornl.gov (J.J.); limy@ornl.gov (Y.C.L.)
† Author note: Charles David Warren retired since Feb 2020

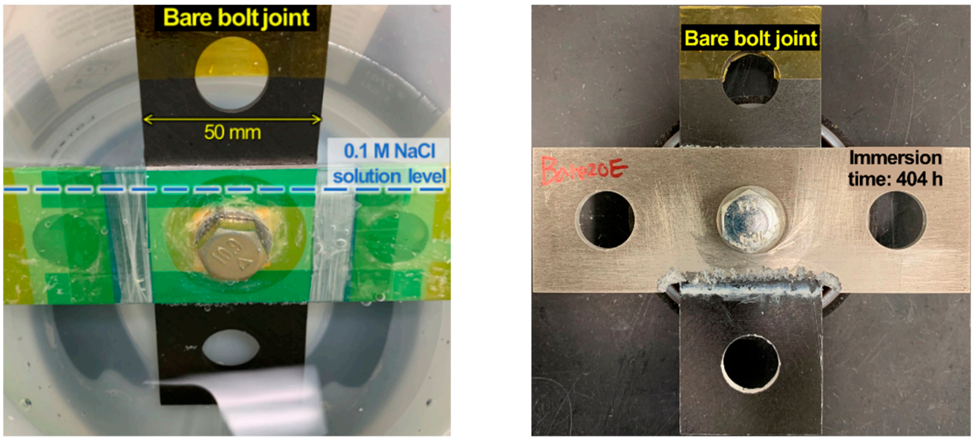

(**a**) (**b**)

Figure S1. Photos of (**a**) a bare bolted joint with tape masking immersed in 0.1 M NaCl, and (**b**) another bare bolt joint that formed corrosion volume in the bottom of the AZ31B sheet after an immersion test for 404 h. Please note that tape masking was removed for the specimen shown in (**b**). The concept of unidirectional growth of corrosion volume in the AZ31B sheet is similar to the artificial pit growth of stainless steels introduced previously [1,2].

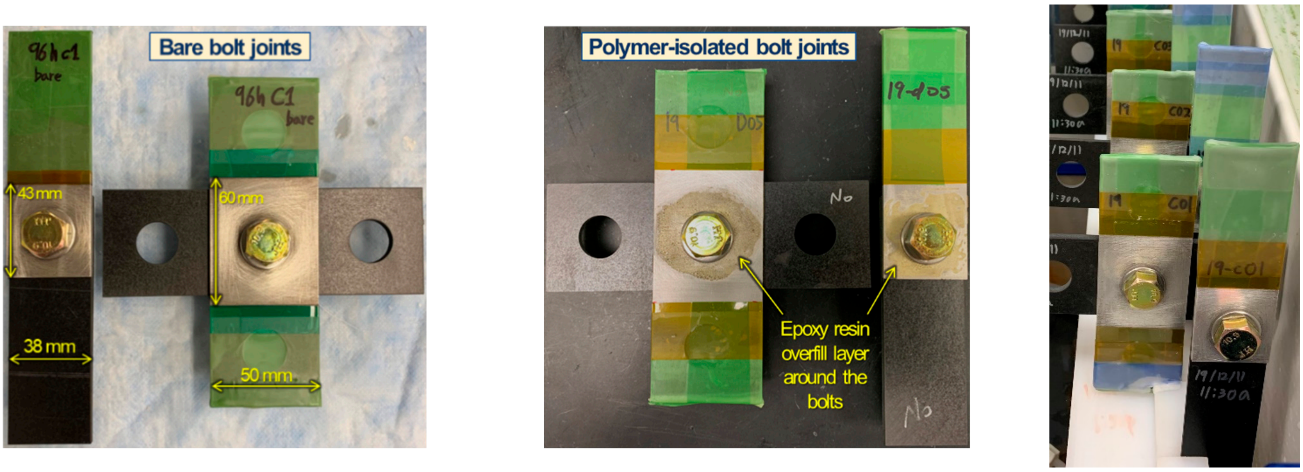

(**a**) (**b**) (**c**)

Figure S2. Photos of tape-masked lap and cross-tension joints in (**a**) bare and (**b**) polymer-insulated conditions, and (**c**) tape-masked bolted joints loaded on a polymer rack inside a salt spray chamber.

Table S1. Chemical analysis of the tap water used to prepare 5 wt.% NaCl solution and produce steam for salt spray exposure test in this work [3,4].

Properties and Concentrations of Chemical Species	Values
pH	7.93
Resistance, Mohm	3.7×10^{-3}
Calcium hardness, as $CaCO_3$, mg/L	79
Iron, as Fe, mg/L	0.06
Copper, as Cu, mg/L	0.01
Zinc, as Zn, mg/L	0.03
Sodium, as Na, mg/L	5.8
Potassium, as K, mg/L	1.6
Chloride, as Cl, mg/L	8.6
Sulfate, as SO_4, mg/L	16
Nitrate, as NO_3, mg/L	2.4
Phosphate, as PO_4, mg/L	<0.1
Silica, as SiO_2, mg/L	5.6

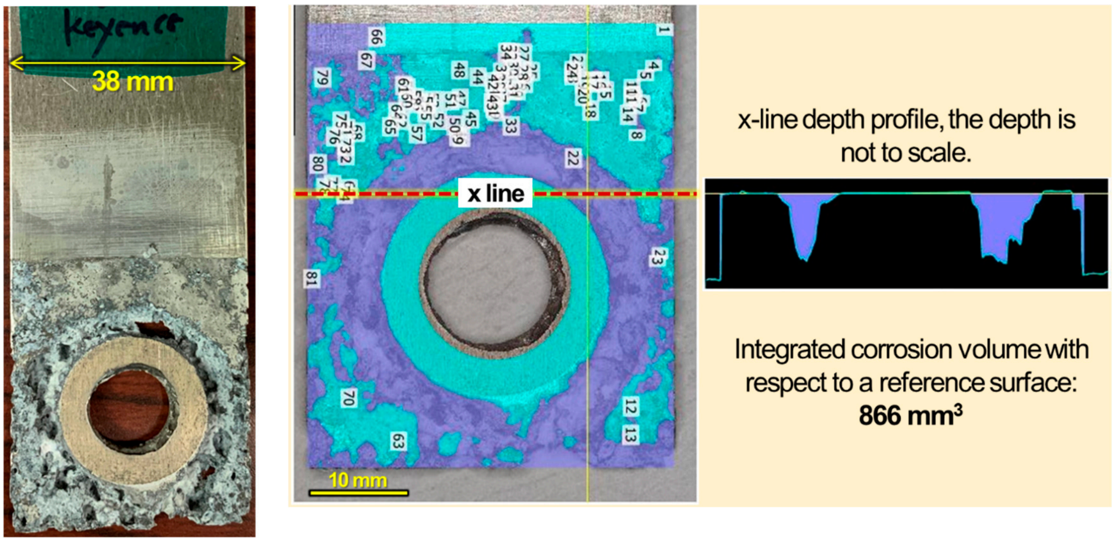

(**a**) (**b**)

Figure S3. Example optical profilometry measurement: (**a**) a AZ31B sheet removed from a bare bolted lap joint after 358 h of salt spray exposure, and (**b**) area and linear depth profiles of the sheet with respect to the uncorroded reference surface. The integrated corrosion volume was 866 mm^3.

References

1. Jun, J.; Frankel, G.S.; Sridhar, N. Effect of chloride concentration and temperature on growth of 1D Pit. *J. Solid State Electrochem.* **2015**, *19*, 3439–3447, doi:10.1007/s10008-015-2780-4.
2. Jun, J. Localized Corrosion of Super 13Cr Stainless Steel in 1D Pit for H2S-free and Sour Brines at An Elevated Temperature. PhD Thesis, The Ohio State University, Columbus, OH, USA 2016.
3. Jun, J.; Sabau, A.; Burns, Z.M.; Stephens, M.S. Corrosion performance of MIL-PRF-23377 primer coated on laser-interference structured aluminum alloy 2024. In Proceedings of the 2019 DoD-Allied Nations Technical Corrosion Conference, Oklahoma City, OK, USA, 12–15 August 2019.
4. Sabau, A.; Jun, J.; Burns, Z.M.; Stephens, M.S. Corrosion resistance of laser-interference structured aluminum alloy 2024 coated with MIL-PRF-85582 primer. In Proceedings of the 2019 DoD-Allied Nations Technical Corrosion Conference, Oklahoma City, OK, USA, 11–15 August 2019.
